# Albumin Reduces Oxidative Stress and Neuronal Apoptosis via the ERK/Nrf2/HO-1 Pathway after Intracerebral Hemorrhage in Rats

**DOI:** 10.1155/2021/8891373

**Published:** 2021-02-24

**Authors:** Shuixiang Deng, Shengpeng Liu, Peng Jin, Shengjie Feng, Mi Tian, Pengju Wei, Hongda Zhu, Jiaying Tan, Feng Zhao, Ye Gong

**Affiliations:** ^1^Department of Critical Care Medicine, Huashan Hospital, Fudan University, Shanghai 200040, China; ^2^Department of Pediatrics, Shenzhen People's Hospital, The Second Clinical Medical College of Jinan University, Shenzhen, Guangdong, China; ^3^State Key Laboratory of Medical Neurobiology and MOE Frontiers Center for Brain Science, Institutes of Brain Science, Fudan University, Shanghai 200032, China; ^4^Department of Neurosurgery, Huashan Hospital, Fudan University, Shanghai 200040, China

## Abstract

**Background:**

Albumin has been regarded as a potent antioxidant with free radical scavenging activities. Oxidative stress and neuronal apoptosis are responsible for its highly damaging effects on brain injury after intracerebral hemorrhage (ICH). Here, the present study investigated the neuroprotective effect of albumin against early brain injury after ICH and the potential underlying mechanisms.

**Methods:**

Adult male Sprague-Dawley rats were subjected to intrastriatal injection of autologous blood to induce ICH. Human serum albumin was given by intravenous injection 1 h after ICH. U0126, an inhibitor of extracellular signal-regulated kinase (ERK1/2), and ML385, an inhibitor of nuclear factor-E2-related factor 2 (Nrf2), were intraperitoneally administered 1 h before ICH induction. Short- and long-term neurobehavioral tests, western blotting, immunofluorescence staining, oxidative stress evaluations, and apoptosis measurements were performed.

**Results:**

Endogenous expression of albumin (peaked at 5 days) and heme oxygenase 1 (HO-1, peaked at 24 h) was increased after ICH compared with the sham group. Albumin and HO-1 were colocalized with neurons. Compared with vehicle, albumin treatment significantly improved short- and long-term neurobehavioral deficits and reduced oxidative stress and neuronal death at 72 h after ICH. Moreover, albumin treatment significantly promoted the phosphorylation of ERK1/2; increased the expression of Nrf2, HO-1, and Bcl-2; and downregulated the expression of Romo1 and Bax. U0126 and ML385 abolished the treatment effects of albumin on behavior and protein levels after ICH.

**Conclusions:**

Albumin attenuated oxidative stress-related neuronal death may in part via the ERK/Nrf2/HO-1 signaling pathway after ICH in rats. Our study suggests that albumin may be a novel therapeutic method to ameliorate brain injury after ICH.

## 1. Introduction

Intracerebral hemorrhage (ICH) constitutes approximately 10% to 20% of all stroke types [1–3] and remains a serious public health problem with high morbidity and mortality [4, 5]. Currently, effective treatment for ICH is lacking due to its multiple injury mechanisms [6–8]. Oxidative stress (OS) and neuronal apoptosis are considered key devastating processes in the pathogenesis of brain injury following ICH [9–11]. Therefore, attenuating OS and neuronal apoptosis may be potential therapeutic targets to improve the prognosis of patients with ICH.

Albumin is a unique pleiotropic protein with a single 585-amino acid polypeptide chain [12]. Albumin is a multifunctional protein that participates in the regulation of colloid osmotic pressure, transportation of endogenous ligands and drugs, and regulation of microvascular permeability [13–15]. Furthermore, albumin has been regarded as a potent antioxidant with free radical scavenging activities and a unique biochemical structure [16, 17]. Previous studies demonstrated that human serum albumin treatment had neuroprotective effects on focal and global cerebral ischemia [18, 19], subarachnoid hemorrhage [20, 21], and ICH [22]. Our previous quantitative proteomics study showed that albumin increased after ICH [23], but the specific mechanism remains unclear.

Extracellular signal-regulated kinase (ERK) 1/2, a prototypic subfamily of MAPKs, is considered the top biological factor that participates in oxidative and ER stress [23]. Albumin therapy activates the downstream protein ERK1/2 signaling [24, 25]. Previous studies showed that nuclear factor erythroid-related factor 2 (Nrf2) is activated by phosphorylate-ERK1/2 (p-ERK1/2) [26], and Nrf2 then enters the nucleus to promote the transcription of the antioxidant-responsive element-regulated gene heme oxygenase-1 (HO-1), which plays a protective role in cellular defense against OS after ICH [27–29]. However, the antioxidation effect of albumin and the potential underlying mechanism after ICH have not been investigated.

The present study was aimed to explore the neuroprotective effect of albumin after ICH. Albumin treatment can suppress OS injury and neuronal apoptosis at least in part via the ERK/Nrf2/HO-1 signaling pathway, thereby improving short- and long-term neurological outcomes after ICH in rats.

## 2. Materials and Methods

### 2.1. Animals

The study was approved by the Animal Care Guidelines of the Animal Experimental Committee of Huashan Hospital Fudan University. Male Sprague-Dawley rats weighing 250-300 g were housed under a 12 h light/dark cycle and had free access to water and food in a humidity- and temperature-controlled environment. Rats were randomly assigned into the experimental and control groups following the random number table.

### 2.2. Experimental Design

**Figure 1:**
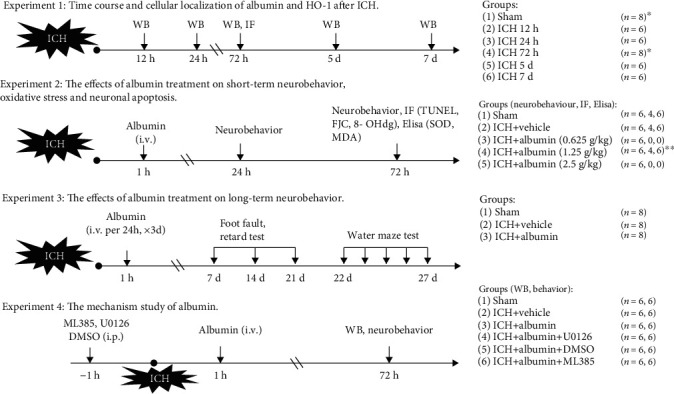
Experimental design and animal groups. ICH: intracerebral hemorrhage; WB: western blot; IF: immunofluorescence; TUNEL: terminal deoxynucleotidyl transferase dUTP nick end labeling; FJC: fluoro-Jade C staining; 8-OHdG: 8-hydroxy-2-deoxyguanosine; MDA: malondialdehyde; SOD: superoxide dismutase; DMSO: dimethyl sulfoxide; i.v.: intravenous injection; i.p.: intraperitoneal injection. ^∗^Extra 2 rats were used for IF (albumin and HO-1 with neurons) at 72 h after ICH. ^∗∗^Assumed the optimal dose group.

A total of four separate experiments were performed, and experimental groups were established (Figure 1). Neurobehavioral functional evaluation and histological images were conducted in a blinded manner.

### 2.3. Experiment 1

To study the endogenous expression of albumin and HO-1 after ICH by western blot, 36 mice were randomly assigned to 6 groups (*n* = 6/group): sham and ICH after 12 h, 24 h, 72 h, 5 d, and 7 d. Additionally, 4 mice were equally distributed to the 72 h post-ICH and sham groups (*n* = 2/group) to conduct double immunofluorescence (IF) staining to assess the localization of albumin and HO-1 on neurons.

### 2.4. Experiment 2

To determine the neuroprotective effects of albumin, 30 rats were randomly divided into 5 groups for neurobehavioral evaluation (*n* = 6/group): sham, ICH+vehicle (saline), ICH+albumin (0.625 g/kg), ICH+albumin (1.25 g/kg), and ICH+albumin (2.5 g/kg). Albumin was given by intravenous injection (IV) at 1 h following ICH. Corner turn, modified Garcia, and forelimb placement tests were used to assess neurobehavioral functions at 24 h and 72 h post-ICH. Based on neurological test results, an additional 30 rats were randomly divided into the sham, vehicle, and albumin (1.25 g/kg) groups (*n* = 10/group). Fluoro-Jade C (FJC) staining, terminal deoxynucleotidyl transferase dUTP (TUNEL) staining, immunofluorescence staining, and malondialdehyde (MDA) and superoxide dismutase (SOD) measurements were used to evaluate the effects of albumin on OS and neuronal apoptosis at 72 h post-ICH. Albumin at the best dose (1.25 g/kg) was administered in experiments 3 and 4.

### 2.5. Experiment 3

To conduct a long-term outcome evaluation, 24 rats were randomly assigned into 3 groups (*n* = 8/group): sham, ICH+vehicle, and ICH+albumin (1.25 g/kg). Albumin was given 1 h post-ICH by IV and then every 24 h for three sequential days. Long-term neurobehavioral tests and the Morris water maze were performed.

### 2.6. Experiment 4

To investigate the neuroprotective mechanism of the ERK/Nrf2/HO-1 pathway, 72 rats were divided into 6 groups (*n* = 12/group): sham, ICH+vehicle (saline), ICH+albumin (1.25 g/kg), ICH+albumin (1.25 g/kg)+DMSO, ICH+albumin (1.25 g/kg)+U0126, and ICH+albumin (1.25 g/kg)+ML385. The ERK1/2 inhibitor U0126 and Nrf2 inhibitor ML385 were administered intraperitoneally (i.p.) at 1 h before ICH. Neurobehavioral tests and western blotting were performed at 72 h post-ICH.

### 2.7. ICH Models

ICH was induced by the double-injection method described for rats [30]. Rats were anesthetized with a mixture of ketamine (100 mg/kg) and xylazine (10 mg/kg). Then, the rate was fixed on a stereotactic frame (David Kopf Instruments) in a prone position. A 1.0-mm burr hole was introduced using a drill in the skull (1.5 mm anterior to bregma, 3 mm lateral to midline, 6 mm below the surface of the skull). A total of 100 *μ*L of fresh autologous blood was taken from the rodent's femoral artery. Thirty microliters of blood was delivered at a rate of 3 *μ*L/min with a microinjection pump (KDS-100, kDa Scientific). After a waiting period of 7 min, 70 *μ*L of blood was delivered to the right striatum at 5 *μ*L/min. Then, the injection cannula was slowly withdrawn 10 min after the second injection. Sham rats underwent similar surgical procedures with only the cannula no blood injection.

### 2.8. Drug Administration

Human serum albumin (25% solution, Baxter Healthcare Corp) was administered via the IV route at 1 h after ICH induction as previously reported [19]. U0126 (sc-222395, Santa Cruz Biotechnology, USA) [31] and ML385 (30 mg/kg) (6887, Tocris, USA) [32] diluted in 5% dimethyl sulfoxide (DMSO) were intraperitoneally administered 1 h before ICH induction.

### 2.9. Short-Term Neurological Performance

Acute neurological deficits at 24 h and 72 h after ICH were assessed in a blinded fashion by an investigator. The corner turn, forelimb placement, and modified Garcia tests were performed as previously described [33]. For the corner turn test, a score was calculated as the number of left turns/10 trials (total) × 100%. For the forelimb placement test, the left forelimb placement was calculated as the number of left forelimb placements/(total forelimb placements) × 100%. The modified Garcia test included 7 scoring systems with a score ranging from 0 to 21.

### 2.10. Long-Term Neurobehavioral Performance

The rotarod and foot-fault tests were performed at days 7, 14, and 21 post-ICH, and the water maze tests were performed at days 22-27 after ICH as previously described [34]. For the water maze testing, the swim distance and escape latency were recorded from testing days 1 to 5 by a video tracking system. The probe test was also recorded by video tracking on testing day 6.

### 2.11. Western Blotting Analysis

Western blotting was performed as previously described [5]. In brief, rats were anesthetized with isoflurane at the established time points after ICH and transcardially perfused with cold PBS. The right brain hemisphere was homogenized in RIPA lysis buffer (sc-24948, Santa Cruz Biotechnology, USA) and further centrifuged at 14000 rpm for 30 min. Equal amounts of protein (4 *μ*L, 30 *μ*g) were loaded onto a 10% SDS-PAGE gel. After separation, the proteins were transferred to a nitrocellulose membrane, which was further blocked for 2 h with a blocking solution. The membranes were incubated with the following primary antibodies overnight at 4°C: anti-albumin (1 : 500, ab207327, Abcam, USA), anti-p-ERK (1 : 1000, Santa Cruz Biotechnology, USA), anti-ERK (1 : 1000, sc-514302, Santa Cruz Biotechnology, USA), anti-Nrf2 (1 : 1000, GTX103322, Gene Tex), anti–HO-1 (1 : 1000, ab68477, Abcam, MA, USA), anti-Romo1 (1 : 1000, Aviva Systems Biology, San Diego, CA), anti-Bcl2 (1 : 1000, ab59348, Abcam, MA, USA), and anti-Bax (1 : 1000, Littleton, CO). The membranes were incubated with an anti-*β*-actin antibody (1 : 5000, sc-47778, Santa Cruz Biotechnology, TX, USA).) The results were normalized using *β*-actin as a loading control. The relative density of the blot bands was quantified by densitometry using the ImageJ software.

### 2.12. Histological Analysis

Rats were anesthetized with isoflurane and transcardially perfused with PBS followed by 100 mL of 4% paraformaldehyde (PFA) as previously described [35]. The brains were rapidly removed and fixed in formalin for 24 h and then dehydrated with 30% sucrose for 3 days. Coronal brain sections (10 *μ*m) were cut using a cryostat (CM3050S, Leica Biosystems, USA) and prepared for double immunofluorescence [36], FJC staining [37], TUNEL staining [37], and 8-hydroxy-2′-deoxyguanosine (8-OHdG) staining [35]. The slides were observed by a fluorescence microscope (DMi8, Leica Microsystems, USA).

### 2.13. Immunofluorescence Staining

Double immunofluorescence staining was conducted at 72 h after ICH. The slides were incubated at 4°C overnight with the following primary antibodies: anti-albumin (1 : 100, ab207327, Abcam, USA), anti-HO-1 (1 : 100, ab68477, Abcam, MA, USA), and anti-NeuN (1 : 150, ab104224, Abcam, Cambridge, MA, USA). The sections were incubated with fluorescence-conjugated secondary antibodies (1 : 200, Jackson ImmunoResearch, USA) at room temperature for 1-2 h before DAPI staining was performed, and sections were visualized and photographed with a microscope.

### 2.14. Fluoro-Jade C Staining

FJC staining was performed to evaluate the number of degenerating neurons with a modified FJC Ready-to-Dilute Staining Kit (Biosensis, Thebarton, South Australia) at 72 h after ICH. According to the manufacturer's instructions and after being washed with PBS, the sections were incubated with the FJC working solution for 20 min and then observed with a fluorescence microscope. The number of FJC-positive neurons was counted in the perihematomal area on three random brain sections per rat over a microscopic field of 400x magnification. The average number of FJC-positive neurons was calculated with the ImageJ software.

### 2.15. TUNEL Staining

To quantify neuronal apoptosis events, double staining with terminal deoxynucleotidyl transferase dUTP nick end labeling (TUNEL) and the neuron marker NeuN was conducted with an In Situ Cell Death Detection Kit with Fluorescein (Roche, Germany) at 72 h after ICH according to the manufacturer's instructions. The number of TUNEL-positive neurons was counted in the perihematomal area. Three random brain sections per slice were used for the mean under a microscopic field of 400x magnification by an independent observer. Data were calculated as the ratio of TUNEL-positive neurons (%) to total neurons.

### 2.16. 8-OHdG Immunohistochemistry

Assessments of brain OS DNA damage and mitochondrial superoxide levels were performed as previously described [35]. In brief, freshly frozen 10-*μ*m-thick brain sections were prepared on normal poly-L-lysine-coated slides. The slides were immersed in antigen retrieval solution (pH 6.0) and heated for 15 min in a microwave to unmask antigens. Then, 3% H_2_O_2_ was added to block endogenous peroxidase for 10 min. The slices were further incubated with an 8-OHdG antibody (1 : 200, ab62623, Abcam, USA) at room temperature. Three randomly selected slices were observed with a microscopic field of 400x magnification. The fluorescence intensity was calculated by the ImageJ software.

### 2.17. Assessment of Intracellular MDA and SOD Levels

To assess intracellular malondialdehyde (MDA) and superoxide dismutase (SOD) activity, perihematomal tissues from each group were collected and homogenized at 72 h post-ICH as previously reported [37, 38]. Commercial MDA kits from Sigma-Aldrich (St. Louis, MO) and an SOD assay kit (Cayman Chemical Company, Ann Arbor, MI, USA) were used in accordance with the manufacturer's instructions. In brief, after cultivation at 95°C for 60 min and cooling in an ice bath for 10 min, 200 *μ*L of the mixed samples was collected into a 96-well plate for analysis with a microplate reader (Bio-Rad, Hercules, CA). Data are presented as micromoles per milligram of protein (*μ*mol/mg protein).

### 2.18. Statistical Analysis

All data are presented as the mean ± standard deviation (mean ± SD). WB data were analyzed by one-way ANOVA followed by Tukey's post hoc test to compare among multiple groups. Two-way repeated measures ANOVA and Tukey's post hoc test were used to analyze behavioral data. *p* values < 0.05 were defined as statistically significant. Statistical analysis was graphed and analyzed by the GraphPad Prism 7 software (La Jolla, CA, USA).

## 3. Results

### 3.1. Animal Use and Mortality

In total, 196 male Sprague-Dawley rats (weighing 250-300 g) from Shrek Company (Shanghai, China) were used for the study. No rats died during the experimental period.

### 3.2. Expression of Endogenous Albumin and HO-1 Increased after ICH

**Figure 2:**
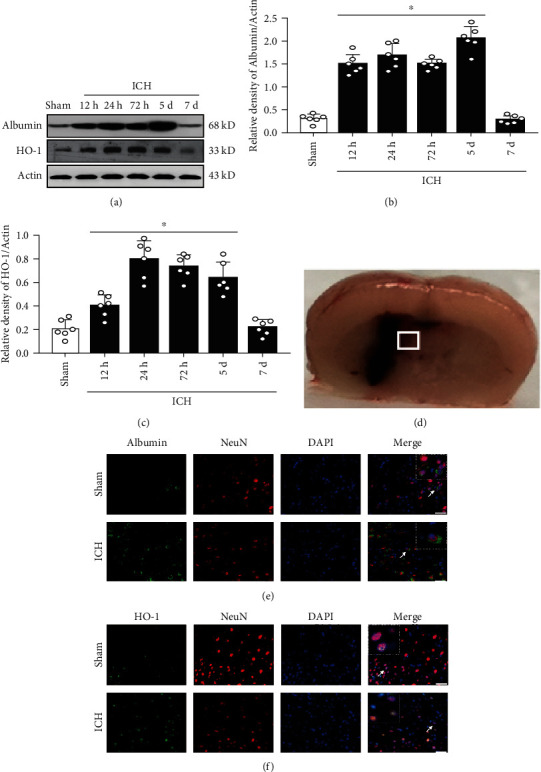
Time course of albumin and HO-1 expression as well as cellular localization of neurons after ICH. (a) Representative WB bands and (b, c) quantitative analysis of albumin and HO-1 expression after ICH; ^∗^*p* < 0.05 vs. sham, mean ± SD, one-way ANOVA, Tukey′s test, *n* = 6/group. (d) Brain sample with schematic illustration showing one area in the perihematomal region and the small white square within the coronal section of the brain indicates the location of where the immunofluorescence staining images were taken. (e, f) Representative microphotographs of coimmunofluorescence staining of albumin (green) with neurons (NeuN, red), as well as HO-1 (green) with neurons (NeuN, red) in the ipsilateral perihematomal region at 72 h after ICH (*n* = 2/group), nuclei were stained with DAPI (blue), scale bar = 50 *μ*m, HO-1: heme oxygenase 1; DAPI: 4′,6-diamidino-2-phenylindole.

Endogenous albumin and HO-1 protein levels were measured by western blot. The expression of albumin increased as early as 12 h after ICH, peaking at 5 days, compared with that in the sham group (*p* < 0.05, Figures 2(a) and 2(b)). HO-1 significantly increased from 24 h to 5 days post-ICH compared with the sham group (*p* < 0.05, Figures 2(a) and 2(c)). Immunofluorescence staining demonstrated that both albumin and HO-1 were colocalized on neurons in the perihematomal region at 72 h after ICH (Figures 2(d)–2(f)).

### 3.3. Albumin Improved Short-Term Neurological Deficits at 24 h and 72 h after ICH

**Figure 3:**
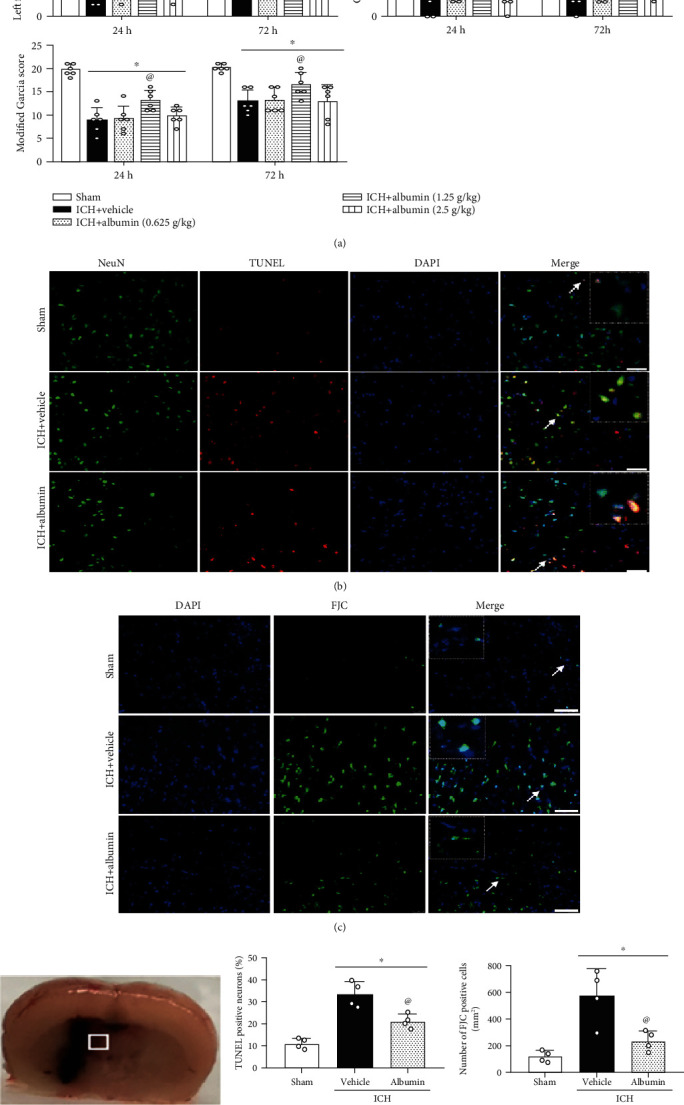
Effects of albumin on short-term neurobehavioral outcome and neuronal damage at 72 h after ICH. (a) Albumin improved short-term neurological function (modified Garcia test, left forelimb placement test, and corner turn test) at 24 h and 72 h after ICH (*n* = 6/group). Short-term neurological results showed that albumin treatment at the medium-dosage of 1.25 g/kg improved short-term neurological function significantly compared to the vehicle and low and high dosage groups. Therefore, we further chose the middle dosage for further study. (b) Representative micrographs of FJC-positive cells within the ipsilateral perihematomal region at 72 h after ICH. (c) Representative micrographs of TUNEL-positive neurons within the ipsilateral perihematomal region at 72 h after ICH. (d) Brain sample with schematic illustration showing one areas in the perihematomal region and the small white square in the coronal section of the brain indicates the area used for counting FJC and TUNEL-positive neurons. (e, f) Quantitative analyses of TUNEL and FJC-positive cells in the perihematomal area at 72 h after ICH (*n* = 4/group); scale bar = 50 *μ*m, nuclei were stained with DAPI (blue). ^∗^*p* < 0.05 vs. sham, ^@^*p* < 0.05 vs. ICH+vehicle, mean ± SD, one-way ANOVA, Tukey's test, TUNEL: terminal deoxynucleotidyl transferase dUTP nick end labeling; FJC: Fluoro-Jade C staining.

The neurobehavioral score was significantly reduced at 24 h and 72 h post-ICH in all ICH groups compared to the sham group (*p* < 0.05, Figure 3(a)). Administration of albumin (1.25 g/kg) significantly ameliorated neurological function at 24 h and 72 h after ICH compared with that in the ICH+vehicle group (*p* < 0.05, Figure 3(a)). The medium albumin dose (1.25 g/kg) was selected for further research based on the neurobehavioral results.

### 3.4. Albumin Reduced Neuronal Apoptosis and Neuronal Degeneration at 72 h after ICH

To determine whether albumin exerts an inhibitory effect on neuronal apoptosis and degeneration, FJC staining and TUNEL staining were performed in the ipsilateral perihematomal area at 72 h after ICH. The numbers of FJC and TUNEL-positive neurons were significantly increased in the ICH+vehicle group compared with the sham group at 72 h after ICH (*p* < 0.05, Figures 3(b)–3(f)). Albumin treatment significantly decreased neuronal apoptosis compared with the ICH+vehicle group at 72 h after ICH (*p* < 0.05, Figures 3(b)–3(f)).

### 3.5. Albumin Reduced Ipsilateral Hemisphere OS Damage at 72 h Post-ICH

**Figure 4:**
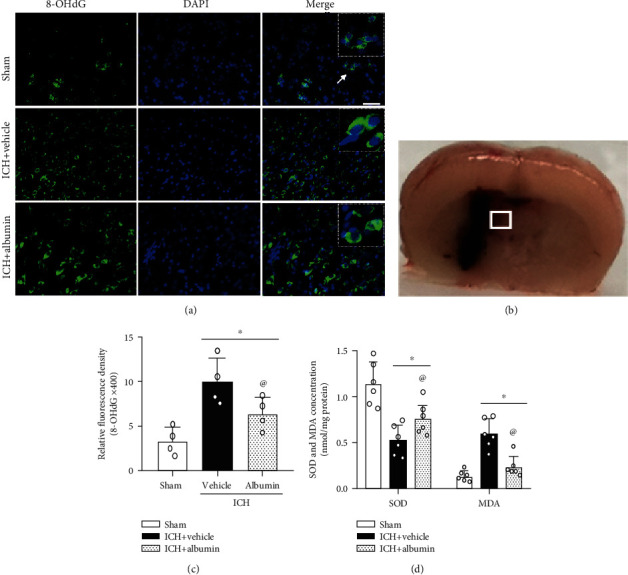
Effects of albumin on oxidative stress levels at 72 h after ICH. (a) Representative micrographs of 8-OHdG (green) immunofluorescence staining in the ipsilateral perihematomal region at 72 h after ICH. Nuclei were stained with DAPI (blue). (b) Brain sample with schematic illustration showing one areas in the perihematomal region and the small white square in the coronal section of the brain indicates the area used for counting 8-OHdG-positive cells. (c) Quantitative analysis of 8-OHdG (green) immunofluorescence staining in the ipsilateral perihematomal region at 72 h after ICH (*n* = 4/group). (d) Superoxide dismutase (SOD) and malondialdehyde (MDA) levels (*n* = 6/group). ^∗^*p* < 0.05 vs. sham, ^@^*p* < 0.05 vs. ICH+vehicle, mean ± SD, one-way ANOVA, Tukey′s test. 8-OHdG: 8-hydroxy-2-deoxyguanosine; SOD: superoxide dismutase; MDA: malondialdehyde.

To investigate the effect of albumin on OS damage post-ICH, we examined the immunofluorescence staining of 8-OHdG (a marker of OS to DNA) and the levels of MDA and SOD in brain tissues on the ipsilateral hemisphere at 72 h after ICH. The fluorescence intensities of 8-OHdG were increased in the ICH+vehicle group compared with the sham group at 72 h after ICH (*p* < 0.05, Figures 4(a)–4(c)). Compared with the ICH+vehicle group, the albumin treatment group exhibited significantly decreased 8-OHdG fluorescence intensity (*p* < 0.05, Figures 4(a)–4(c)). Similar results were also observed regarding MDA and SOD measurements. After ICH induction, the levels of SOD were markedly lower in the ICH+vehicle group than in the sham group (*p* < 0.05, Figure 4(d)), whereas the MDA levels were more pronounced in the ICH+vehicle group than in the sham group at 72 h after ICH (*p* < 0.05, Figure 4(d)). Furthermore, upon administration of albumin, SOD levels increased while MDA levels decreased when compared with the levels in the ICH+vehicle group (*p* < 0.05; Figure 4(d)).

### 3.6. Albumin Improved Long-Term Neurological Deficits at 28 d after ICH

**Figure 5:**
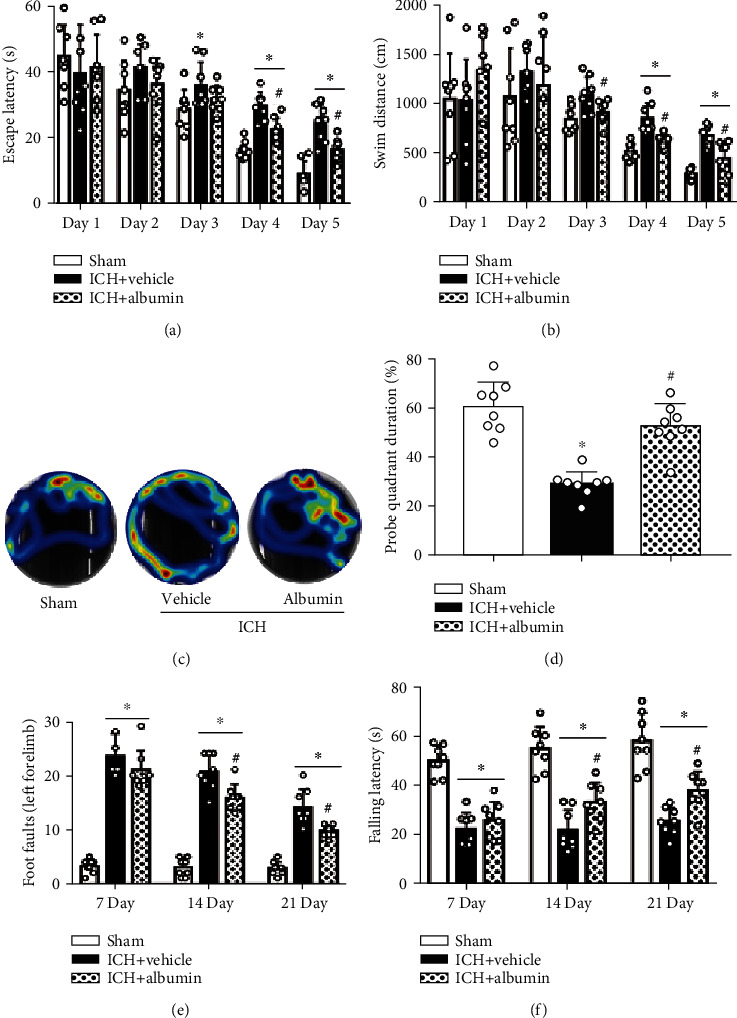
Albumin (1.25 g/kg, i.v.) treatment improved long-term motor and memory function after ICH. (a–d) Water maze tests performed from days 22-27 after ICH, (e) foot fault test, and (f) rotarod test performed at days 7, 14, and 21 after ICH. ^∗^*p* < 0.05 vs. sham, #*p* < 0.05 vs. ICH+vehicle, mean ± SD, two-way repeated measures ANOVA (a, b), one-way ANOVA for (d–f), Tukey′s test, *n* = 8/group.

The Morris water maze test revealed that after ICH surgery, memory of space and the ability to learn were more damaged in the ICH+vehicle group than in the sham group during testing days 3 to 5, leading to a prolonged escape latency and much longer swim distance as well as less time spent in the probe quadrant (*p* < 0.05; Figures 5(a)–5(d)). Compared with the ICH+vehicle group, the albumin treatment group had significantly improved performance with a shorter escape latency and swimming distance as well as more time spent in the probe quadrant on days 3 to 5 (*p* < 0.05; Figures 5(a)–5(d)). In addition, the rotarod test at days 7, 14, and 21 after ICH showed that ICH+vehicle rats exhibited remarkedly persistent neurological dysfunctions compared with those in the sham group, and ICH+vehicle rats displayed a shorter falling latency than those in the sham group (*p* < 0.05; Figures 5(e) and 5(f)). Rats in the albumin treatment group showed significant improvement in both tests (*p* < 0.05; Figures 5(e) and 5(f)).

### 3.7. Albumin Attenuated OS and Neuronal Apoptosis via the ERK/Nrf2/HO-1 Signaling Pathway at 72 h after ICH

**Figure 6:**
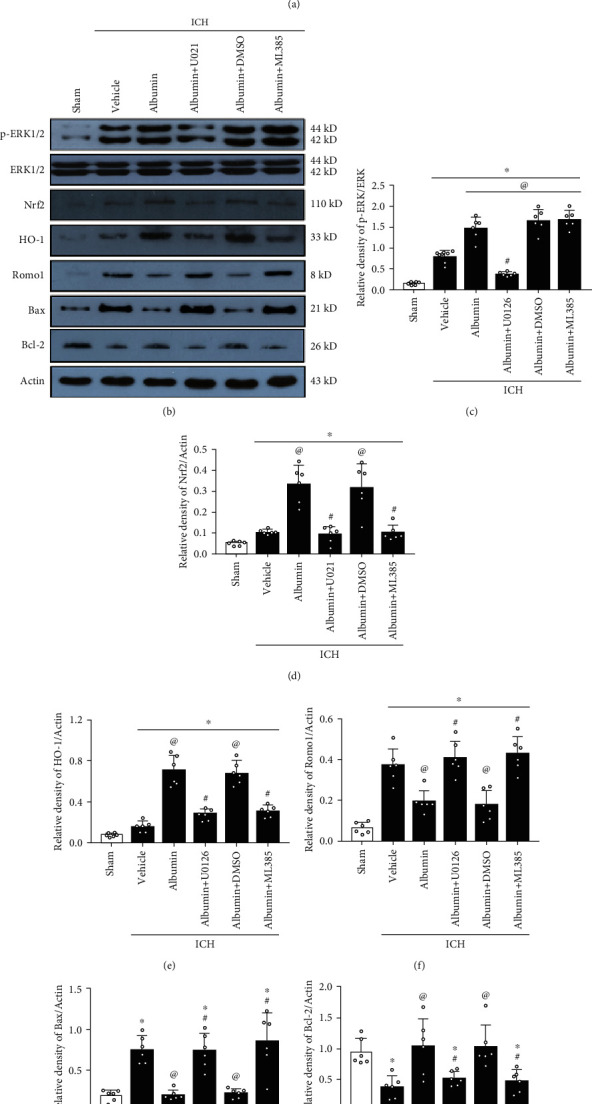
Blockade of ERK and Nrf2 reversed the effects of albumin on short-term neurobehavioral outcome and oxidative stress-induced neuronal death at 72 h after ICH. (a) Modified Garcia test, left forelimb placement test, and corner turn test. (b) Representative western blotting images. (c–h) Quantitative analyses of p-ERK/ERK, Nrf2, HO-1, Romo1, Bax, and Bcl-2 expression. ^∗^*p* < 0.05 vs. sham, ^@^*p* < 0.05 vs. ICH+vehicle, ^&^*p* < 0.05 vs. ICH+albumin, ^#^*p* < 0.05 vs. ICH+albumin+DMSO; mean ± SD, one-way ANOVA, Tukey's test, *n* = 6/group. p-ERK1/2: phosphorylated extracellular regulated protein kinases 1/2; ERK1/2: extracellular regulated protein kinases1/2; Nrf2: nuclear factor-E2-related factor 2; HO-1: heme oxygenase 1; Romo1: reactive oxygen species modulator 1; Bax: B-cell lymphoma-2 associated X protein; Bcl-2: B-cell lymphoma-extra large 2.

U0126 and ML385 reversed the neuroprotective effects of albumin on neurobehavioral outcomes after ICH (Figure 6(a)). Western blots were conducted to assess albumin downstream signaling molecules and markers of OS and neuronal apoptosis at 72 h after ICH. The expression of p-ERK, Nrf2, HO-1, and Bcl-2 was significantly increased, but that of Romo1 and Bax was decreased in the ICH+albumin group compared with the ICH+vehicle group (*p* < 0.05, Figures 6(b)–6(h)). The inhibition of ERK1/2 by U0126 with albumin administration significantly decreased the levels of downstream proteins, including p-ERK, Nrf2, HO-1, and Bcl-2, in the ICH+albumin+U0126 group compared with the ICH+albumin and ICH+albumin+DMSO groups (*p* < 0.05; Figures 6(b)–6(e) and 6(h)). Consistently, significant increases in Romo1 and Bax expression were observed in the ICH+albumin+U0126 group compared to the ICH+albumin and ICH+albumin+DMSO groups (*p* < 0.05; Figures 6(f) and 6(g)).

In addition, the increased expression of p-ERK after ICH was not changed by the administration of albumin and ML385 (Figures 6(b) and 6(c)). Nrf2 knockdown by ML385 pretreatment significantly decreased the expression of Nrf2, HO-1, and Bcl-2 but increased the protein levels of Romo1 and Bax at 72 h after ICH in the ICH+albumin+ML385 group compared with the ICH+albumin and ICH+albumin+DMSO groups (*p* < 0.05; Figures 6(b) and 6(d)–6(h)).

## 4. Discussion

In the present study, we assessed the effects of albumin on OS and neuronal apoptosis after ICH in a rat model and investigated the mechanism underlying the effects of albumin after ICH. For the first time, we indicated that the endogenous protein levels of albumin and HO-1 were increased after ICH in rats and peaked at 5 days and 24 h, respectively, after ICH. Albumin and HO-1 were colocalized on neurons at 72 h after ICH. In addition, medium-dose albumin therapy significantly ameliorated the short- and long-term neurological deficits and alleviated OS damage and neuronal apoptosis after ICH. Mechanistically, albumin administration increased the protein levels of p-ERK1/2, Nrf2, HO-1, and Bcl-2 and decreased the protein levels of Romo1 and Bax within the ipsilateral perihematomal area at 72 h after ICH. Inhibitor of ERK1/2 or Nrf2 reversed the beneficial effects of albumin on neurological deficits, OS, and neuronal apoptosis. These findings indicate that albumin administration possibly attenuates OS and neuronal apoptosis of brain injury, thereby improving in neurobehavioral deficits after ICH in rats. This neuroprotection was attributed at least in part to the ERK/Nrf2/HO-1 signaling pathway.

OS plays a substantial role in the pathogenesis of neurological diseases [11, 35, 39]. Unlike ischemic stroke and other neurological diseases, a central event of ICH involving OS is the abundant free heme release from red cells into the extracellular space and the metabolism of heme providing catalytically active iron to neighboring tissues, which leads to secondary neuronal damage and apoptosis [10]. Therefore, suppression of OS and neuronal apoptosis is a potential therapeutic tactic for ICH.

Serum albumin is a multifunctional plasma protein that is involved in many physiologic processes and has diverse biological effects [40]. Previous studies have shown that albumin can be regarded as an indicator of nutritional status and a strong predictor of mortality for various acute and chronic illnesses [41–43]. In addition, albumin has a neuroprotective role in neurological diseases, including Alzheimer's disease, cerebral ischemia, and subarachnoid hemorrhage, due to its ability to inhibit polymerization and enhance the clearance of amyloid *β*, regulate hemodynamic properties, and exhibit antithrombotic and anti-inflammatory activities [44]. Ludmila B and his colleagues found that after acute intracortical hematoma, albumin therapy improved neurological function and blood-brain barrier integrity in the acute phase, but they did not elucidate the mechanism of the neuroprotective effect of albumin [22].

In the present study, we broadened the albumin application to the setting of ICH. Albumin has been shown to exhibit antioxidative stress and antiapoptotic effects after ICH as well as direct neuroprotective actions on neuronal cells. First, a marked increase in endogenous albumin levels was observed in the ipsilateral brain hemisphere after ICH. Furthermore, double immunofluorescence staining indicated that albumin was localized in neurons. The following factors could explain the increase in albumin in the brain post-ICH: (a) after ICH, the blood-brain barrier is destroyed, and albumin oozes from the blood vessels into the brain tissue; and (b) brain cells such as microglia may synthesize and secrete albumin, as reported in a Human Brain Proteome Project study [45]. Although albumin is considered an extracellular molecule, we found that it is localized in neurons, which is in accordance with the notion that albumin uptake can occur in many cell types, including neurons, by endocytosis [46].

Second, previous studies have shown that albumin protects against OS and apoptosis in many conditions [47, 48]. In the present study, albumin had a protective effect against OS and apoptosis after ICH. Neuroprotection of albumin after ICH is regulated via multiple mechanisms, including increased serum oncotic pressure to improve brain tissue perfusion, alleviate brain edema, maintain normal endothelial integrity, and exert antioxidant effects [22, 49]. More importantly, albumin therapy might have other beneficial effects distinctive to ICH itself. The particular thiol structure of albumin makes up almost 80% of extracellular thiols, making albumin a major extracellular protein target for OS. Albumin in the extracellular space shows a high affinity for free heme that is released from hemorrhaged or dying red cells to provide a lipid antioxidant effect. Furthermore, albumin also inhibits OS injury by neutralizing free Cu2^+^ and iron, which are released from catalysis reactions [50].

Although albumin has a variety of neuroprotective effects on ICH-induced injury, however, the potential molecular mechanisms involved with the activation of ERK by albumin have not been fully investigated. Our previous quantitative proteomics study [23] showed that the ERK1 and ERK2 cascades were the top two biological processes after ICH and that the albumin and ERK1/2 signaling pathways were the top protein-protein interaction networks. Recently, some studies showed that there was an important link between albumin and the mitogen-activated protein kinase (MAPK) extracellular signal-regulated kinase, ERK1/ERK2 in kidney disease, and ERK1/2 signaling pathway was the downstream signal activated by albumin therapy [24, 51]. Reich et al. found that albumin-activated ERK via EGF receptor in human renal epithelial cells and the generation of ROS after albumin exposure was an important proximate event in the albumin-induced cell signaling [52]. In the present study, albumin application significantly increased the protein levels of p-ERK1/2, and inhibition of ERK1/2 abolished the protective effects of albumin on neurological deficits, OS, and neuronal apoptosis. Therefore, the present findings support the hypothesis that ROS produced after ICH might be the possible reason for albumin-induced activation of the ERK signaling pathway. Our data only provided a link between albumin and the ERK signaling pathway, but further investigation is needed to elucidate the precise mechanism of albumin activating the ERK signaling pathway after ICH. Nrf2 is considered the downstream factor of ERK1/2 activation [53]. Activated Nrf2 orchestrates multiple responses to OS and controls the transcription of antioxidant-responsive element-regulated genes, including HO-1 and SOD, which help to protect cells against OS and apoptosis. Activated Nrf2 also reduced peroxide formation by enhancing antioxidative activity and hematoma resolution after ICH [54]. In our study, albumin application significantly increased the protein levels of Nrf2 and HO-1 in the ipsilateral basal ganglion at 72 h after ICH. Inhibition of Nrf2 abolished the protective effects of albumin on neurological deficits, OS, and neuronal apoptosis. Taken together, these results suggest that albumin administration attenuates OS and neuronal apoptosis after ICH at least in part through the ERK/Nrf2/HO-1 signaling pathway, thereby improving in neurobehavioral deficits after ICH in rats.

There are several limitations in the present study. First, we did not test each protein at the gene level or the expression of albumin in the serum after ICH. Second, previous studies showed that albumin was also expressed in endothelial and astrocyte cells; therefore, we cannot exclude the possibility that the blood-brain barrier protection and anti-inflammatory effects of albumin played a role after ICH. Third, Nrf2 helps upregulate CD163 and CD36 expression in microglia; thus, we cannot exclude the possibility that the protective effect of albumin in promoting hematoma clearance is due to the contributions of other Nrf2 signaling pathways.

## 5. Conclusions

In conclusion, albumin application attenuated oxidative stress-related neuronal death after ICH in rats, thereby improving neurological deficits, and these effects were related at least in part to activation of the ERK/Nrf2/HO-1 signaling pathway. Our study suggests that albumin may serve as a promising strategy for the treatment of ICH.

## Data Availability

The data support the findings of this study and are available from the corresponding author upon reasonable request.
